# Antinociceptive and Smooth Muscle Relaxant Activity of* Croton tiglium *L Seed: An *In-vitro* and *In-vivo* Study

**Published:** 2012

**Authors:** Zhen Liu, Wenyuan Gao, Jingze Zhang, Jing Hu

**Affiliations:** a *School of Pharmaceutical Science and Technology, Tianjin University, Tianjin 300072, China. *; b *School of Chinese Medicine, Tianjin University of TCM, Tianjin 300193, China.*

**Keywords:** Croton tiglium L, Intestinal propulsion, Smooth muscle, Rabbit jejunum, Antinociceptive, Phorbol esters

## Abstract

The seed of* Croton tiglium *L. (SCT) is a well known folk medicine. In China, it has used to treat gastrointestinal disorders, intestinal inflammation, rheumatism, and so on. Previous studies established its purgative and inflammation properties. In addition, the effects of essential oil of SCT on intestinal transit and gastrointestinal tract has been studied. In the present study, we evaluated the antinociceptive effect of SCT through the writhing test in mice, investigated the effects of it on spontaneous smooth muscle contractions of isolated rabbit jejunum and examined the *in-vitro* results through the *in-vivo* small intestine propulsion. We further investigated the possible compounds using HPLC-MS, and six compounds were tentatively identified as phorbol esters. Furthermore, the possible fragmentation pathways of phorbol esters were proposed, and we also detected the possible compounds in the active parts.

## Introduction

The genus *Croton* belongs to the family Euphorbiaceae. The seed of *Croton tiglium* L. (SCT) is well known as Ba-Dou in China. It has been used as a traditional medicine for many applications such as constipation, a purgative, and treating dyspepsia and dysenteria. The Chinese had written records in the second century B.C. for using it to treat the gastrointestinal disorders, intestinal inflammation, rheumatism, headache, peptic ulcer and visceral pain (1-4). Croton oil, the essential oil of SCT, as the effective part, has been reported to have purgative, analgesic, antimicrobial, and inflammatory properties (1, 3). Besides, the direct effect of Croton oil on guinea pig colonic smooth muscle cell has been studied, which regulates the gastrointestinal transit in mice, and affects the inflammatory and immunological milieu (2, 5). In the previous study, we reported the effect of croton oil on spontaneous smooth muscle contractions in isolated rabbit jejunum and the underlying mechanisms (6). From the leaves of *C. tiglium,* a pyrazine derivative crotonine was isolated and showed significance analgesic effects (7).

Some tigliane phorbol esters have been isolated from *C. tiglium* previously. Among *Croton* species, only *C. tiglium* has been extensively studied as the source of phorbol derivatives (8, 9). Phorbol esters have been shown to be responsible for eliciting a remarkable range of biochemical effects except tumor promoting (10, 11), such as skin irritant effects (12), platelet aggregation (13), and cell differentiation (14). Although the ability of these compounds to promote tumors presents the potential limitation of their utility, it should be stressed that there are many phorbol esters that exert the profound beneficial biological effects without tumorigenesis. 12-O-tigloylphorbol-13-decanoate isolated from croton oil demonstrated antileukemia activity against the P-388 leukemia in mice (15). Eight phorbol esters isolated from the *C. tiglium* have the ability to inhibit an HIV-induced cytopathic effect on MT-4 cells (16). The most investigated activity of the phorbol esters is their binding and activation of protein kinase C (PKC), which plays a critical role in signal transduction pathway and regulates the cell growth and differentiation (17, 18). PKC isoforms are distributed in the small intestine and involved in modifying the functions of intestinal muscle including the generation of slow, sustained contraction of smooth muscle cells through Ca^2+^ influx, as shown through the experiments using phorbol esters and isozyme-specific blockade (19, 20). Our previous study proved quite consistent with this (6).

The commonly used models of analgesia contain thermal stimulation, electrical stimulating method, mechanical irritation and chemical stimulus. With different models, there can be differences in the analgesic effects of selected drugs; some trends can be identified (21-22). The writhing test is an experimental model used for the screening of drugs with analgesic activity, based on the irritation caused after the intraperitoneal injection of 0.6% acetic acid. This injection can produce the peritoneal inflammation characterized through the contraction of abdominal muscles accompanying an extension of the forelimbs and elongation of the body. This writhing response is considered to be a visceral inflammatory pain model, and in this way, this acid causes the release of algesic mediators such as bradykinin, prostaglandins, histamine and 5-hydroxytryptamine. Additionally, although this test was a nonspecific model (*e.g*. anticholinergic and antihistaminic and other agents show activity in this test), it is widely used for analgesic screening and involves local peritoneal receptors (23).

So in the present study, we examined the antinociceptive effects of SCT in acetic acid-induced writhing in mice, and investigated the active fraction of SCT on spontaneous smooth muscle contraction in isolated rabbit jejunum. By the results of the muscle contraction, we tested the effect of SCT on intestinal propulsion in mice using the charcoal method. The effective compounds of SCT and the possible fragmentation of phorbol esters are also described in this assay.

## Experimental

*Plant materials and animals*

The seed of *C. tiglium* was provided by Tianjin Lerentang Pharmaceutical Factory (Tianjin, China) and identified by Professor Wenyuan Gao from School of Pharmaceutical Science and Technology, Tianjin University, China. The voucher specimen (voucher No. BD070701) are available in the herbarium of Research Center of Tianjin Zhongxin Pharmaceuticals.

Adult male and female New Zealand white rabbit (2.0-2.5 kg) were obtained from Laboratory Animal Center of Peking University (Beijing, China). All animals were housed at the Experimental Animal Center of Tianjin Medical University (Tianjin, China) and kept under standard environmental conditions. Animals had free access to water, but food was withdrawn 24 h before the experiments. KM mice (18-22 g) were housed in plastic cages, with food and tap water available *ad libitum*, in the colony room. The animals submitted to oral administration of the extract or drugs were fasted for 24 h. 

**Table 1:** Possible compounds from SCTp and SCTe by HPLC-ESI(+)/MS^n^.

**NO**	**Compound**	**Molecular formula**	**[M+H]** ^+^ ** or [M+Na]** ^+^ ** m/z**	**Fragment ions of [M+H]** ^+^ ** or [M+Na]** ^+^
**1**	Deoxyphorbol acetate methylbutanoate	C_27_H_38_O_7_	475.1	355.3, 311.0, 277.0, 293.0, 265.1
**2**	Phorbol acetate methylbutenoate	C_27_H_36_O_8_	489.2	429.3, 389.3, 311.2, 293.0, 265.1
**3**	Deoxyphorbol acetate methylbutenoate	C_27_H_36_O_7_	473.0	391.0, 311.2, 293.1, 265.1
**4**	Phorbol methylbutanoate isobutyrate	C_29_H_42_O_8_	519.6	311.0, 293.0, 265.1
**5**	Phorbol decanoate acetate	C_32_H_48_O_8_	583.3	501.8, 311.1, 293.1, 269.0, 265.1
**6**	Phorbol acetate butyrate	C_26_H_36_O_8_	499.1	417.3, 311.2, 293.1, 265.0

Animals’ experiments were performed with the approval of the Institutional Animals Care and Use Committee of China, and institutional guidelines for animal welfare and experimental conduct were followed.

*Reagents and chemicals*

HPLC-grade acetonitrile was from Merck (Darmstadt, Germany). Acetylcholine (Ach), Hexamethonium, Methoctramine, and 4-Diphenylacetoxy-N-methylpiperiding methiodide (4-DAMP) were purchased from Sigma (St. Louis, MO, USA ) and Verapamil Hydrochloride Injection was obtained from Hefeng Co., Ltd. (Shanghai, China). Atropine sulphate injection and Noradrenaline Bitartrate were supplied by Jinhui amino Co., Ltd. (Tianjin, China). Other chemicals were of the highest grade available.

**Table 2:** Effects of SCT extract on acetic acid-induced abdominal writhing in mice.

Treatment	Dose (mg/Kg)	Acetic acid-induced writhing test	
		Number of writhes (30 min)	Inhibition (%)
Vehicle (p.o*.*)	-	24.5±	-
SCTm (p.o*.*)	25	20.5±	16.3%
	50	17.0±	30.6%
	100	13.3±	45.7%
	200	21.7±	11.4%
	250	23.3±	1.2%
	300	23.8±	2.9%
SCTe (p.o*.*)	20	15.5±	36.7%
SCTp (p.o*.*)	20	15.2±	38.0%
Aspirin (p.o*.*)	100	8.1±	80.0%

Values represent the mean ±SEM of 7 mice.* Signiﬁcantly different from control group: p < 0.05.** Signiﬁcantly different from control group: p < 0.01.

*Extraction of the seed of C. tiglium*

SCT (2 Kg) was extracted with methanol (10L × 3) under reflux for 3 h. The methanol extracts (SCTm) were combined and evaporated under reduced pressure in a rotary evaporator to give an oily residue (250 g), with a yield of 12.5%. The residue was suspended in aqueous and extracted with petroleum ether, ethyl acetate and normal butanol. The extracted solutions were respectively evaporated under reduced pressure to give P.E. parts (SCTp) (167 g), EtOAc parts (SCTe) (8 g), *n*-BuOH parts (44 g) and H_2_O fraction (8 g). Croton oil was the same as previously reported (6).

**Table 3:** Effects of SCT extracts on small intestine propulsion in mice (*x±s*, n = 10).

**Treatment**	**Dose (mg/kg)**	**Transversed (%)**
Control	-	57.94 ± 10.82
SCT	200	75.06 ± 10.53 **
SCTm	50	77.62 ± 12.98 **
SCTp	20	83.23 ± 10.72 **
SCTe	10	100.00 ± 0.00 **

* Signiﬁcantly different from control group: p < 0.05.** Signiﬁcantly different from control group: p < 0.01.

*Writhing test*

This test was done using the method described by Koster *et al*. (25). Seventy male and female KM mice were used in this experiment and they were divided equally into ten groups. Mice were pretreated as follows: Group 1 (Control group), water solution (10 mL/Kg, p.o*.*); Group (2-7), SCTm (25, 50, 100, 200, 250, 300 mg/Kg, p.o*.*); Group 8, SCTp (20 mg/Kg, p.o*.*); Group 9, SCTe (20 mg/Kg, p.o*.*); Group10, Aspirin (100 mg/Kg, p.o*.*). All substances were administered 60 min before the intraperitoneal acetic acid injection (0.6%, 0.1 mL/10 g, IP) and the number of writhes was counted for the following 30 min. All the doses of the different parts were based on the data showed in the previous paper (6).

*Tissue preparation*

Thirty-six rabbits were sacrificed using a blow on the head. After a laparotomy incision, a portion of the jejunum was removed and placed in an oxygenated Tyrode’s solution (composition in mM: NaCl 136.9, CaCl_2_ 1.8, KCl 2.7, MgCl_2_ 1.1, NaHCO_3_ 11.9, NaH_2_PO_4_ 0.4, and glucose 5.6, pH = 7.4). Respective five segments of jejunum 2 cm in length were mounted in a 10 mL organ bath containing Tyrode’s solution that was bubbled with a 95% O_2_ and 5% CO_2_ gas mixture and the temperature was held at 37°С (6).

*Contractile activity of smooth muscle*

Each segment was allowed to equilibrate in the bath for 50 min to obtain a regular spontaneous activity. An initial load of 1 g was applied to each of the tissue and was kept constantly throughout the experiment. The muscle tension, measured with a force transducer (Model JH-2, Beijing, China), was displayed on a multichannel recorder (HV-4, Taimeng, China) and monitored with a Biology BL-410 computer. Then, the following experiments were performed.

Croton oil, SCTm, SCTp and SCTe were separately prepared as 40 mg/mL stock solution in 0.5% tween-80 and additional dilutions were made with distilled water. A single concentration of them (20, 60, 80, 100, 200 μg/mL) was added to the organ bath., *n*-BuOH parts and H_2_O fraction were prepared as 0.5 g/mL stock solution in 0.5% tween-80 and additional dilutions were made with distilled water. The end concentrations were 0.1, 0.5, 1.0, 2.0 and 4.0 mg/mL. In this experiment, each dose that was designed according to the prepared experiment that displayed a turning point used six tissue preparations.

The average peak intensity, tension and frequency of contractions occurred before (5 min) and after (5 min) administration of each drug were determined. Relative changes of drug-induced contractile responses to the basal levels (before the treatment of drugs) were calculated as percentage.

*Small intestinal propulsion*

The effect of SCT on intestinal propulsion in KM mice was tested using the charcoal method (26). Fifty male and female KM mice were fasted for 12 h but allowed free access to water. The animals were randomly allotted into five groups of ten animals per group. Group 1 was administered with distilled water (10 mL/Kg, p.o.) using orogastric cannula. Group 2 was pretreated with *C. tiglium* (200 mg/Kg, p.o.). Group 3 was pretreated with SCTm (50 mg/Kg, p.o.) while group 4 and 5 received SCTp (20 mg/Kg, p.o.) and SCTe (10 mg/Kg, p.o.), respectively. Thirty minutes after the treatment with the extract, each mouse was administered with 0.2 mL of standard charcoal meal (5% activated charcoal suspended in 10% gum acacia) orally. The mice were killed 30 min later through cervical dislocation, and the small intestine was rapidly dissected out and placed on a clean surface. The small intestine was carefully inspected and the distance traversed using the charcoal meal from the pylorus was measured for both the control and treated groups. For each group, the results were expressed as percentage of the distance traveled from the pylorus to the caecum (27).

*HPLC-MS and ESI-MS* ^n^ *of P.E. parts and EtOAc parts of C. tiglium*

SCTp and SCTe were analyzed through high-pressure liquid chromatograms (HPLC)-mass spectrometer (MS). Briefly, the extracts were analyzed via HPLC-mass spectrometer using HPLC (Agilent technologies 1200 series Diode Array detector) with an ion-trap ESI-mass spectrometer. Samples were injected into a Kromasil RP-C_18_ column (4 mm×250 mm). The column was equilibrated in water (solution A) and elution of the components was achieved by increasing the concentration of solution B (100% acetonitrile) from 5 to 95% in 60 min at a flow rate of 0.8 mL/min. The molecular masses of the peaks were determined from electro-spray ionization mass spectra using multiply-charged ion profile.

The Agilent HPLC-MS system contains a survey or auto-sampling system, interfaced to an ion-trap mass spectrometer via an electro-spray ion source. Source setting used for analysis the extracts were: nebulizer gas flow, 30.00 psi; dry gas flow, 8.00 L/min; capillary temperature, 350°C; Nitrogen (> 99.99%) and He (> 99.99%) were used as sheath and damping gas, respectively.

The full scan of ions ranging from m/z 100 to 1,000 in the positive ion mode was carried out. The fragment ions were obtained for both MS^2 ^and MS^3^ experiments. Analyses were conducted at ambient temperature and the data were operated on the Xcalibur software.

*Statistical analysis*

Data were expressed as mean SEM with *n* denoting the number of tested tissue preparations. SPSS 15.0 (for Windows) was used to analyze the data. Student’s t-test was used to analyze and compare the results between the groups while a one-way ANOVA was used to compare the results among the groups. Differences were considered statistically significant if p < 0.05.

## Results and Discussion

*The result of writhing test in mice*

In the writhing test in mice, SCTm at doses of 25, 50 and 100 mg/Kg, inhibited the frequency-induced abdominal constrictions with acetic acid in a dose-dependent manner and results were statistically significant (Table 2). The results obtained for SCTm, SCTp and SCTe were similarly and all showed weak analgesic effect when compared to aspirin (positive control, 100 mg/Kg, 80.0%). The oral pre-treatment of mice with SCTm (100 mg/Kg), SCTp (20 mg/Kg) and SCTe (20 mg/Kg) resulted in a 45.7%, 38.0% and 36.7% inhibition of the abdominal writhing, respectively.

**Figure 1:**
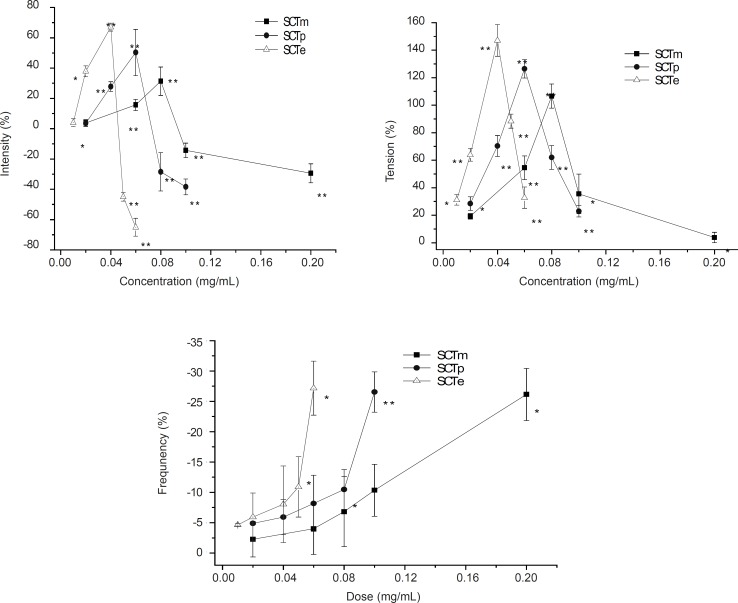
Representative intensity (A) and tension (B) or Frequency (C) on the smooth muscle contractions in isolated rabbit jejunum induced by SCTm, SCTp and SCTe with five different doses (0-0.2 mg/mL). The dates were measured by isometric force transducers before (5 min) and after (5 min) treatment of each parts. Each point represents mean ± SEM. of six tissues (n = 6). ^*^ p < 0.05^ **^, p < 0.01 compared to the corresponding values of basal contractility.

*Effects of the different parts of C. tiglium on spontaneous smooth muscle contractions*

The method used in the experiment is the same as previously reported (6). We observed that the action of SCTm was concord with that of Croton oil, but with a better effect in muscle contraction. SCTp and SCTe increased the activity of muscle contraction, and the tendency of the effect was the same as SCTm, which at the low concentrations, concentration-dependency increased the amplitude and tension of muscle contractions and at high concentration, it decreased the intensity and tension of muscle contractions. In addition, they suppressed the frequency of muscle contractions in a concentration-dependent manner. At the concentrations of 40 μg/mL, SCTe reached the maximum, whereas, SCTp and SCTm reached the maximum at the concentrations of 60 μg/mL and 80 μg/mL (Figure 1). However, *n*-BuOH parts and H_2_O fraction decreased this effect.

*Effect on small intestine propulsion*

For testing the results of SCT in muscle contraction *in-vitro*, we examined the effect on small intestinal propulsion *in-vivo*. In control animals, at 30 min after the intragastric administration, the charcoal meal transversed 57.94% of the total length of the small intestine (Table 3). SCT, SCTm, SCTp and SCTe promoted small intestinal transit progressively (75.06-100.00%, p < 0.01). This result consisted with the result *in-vitro* testing.

**Figure 2:**
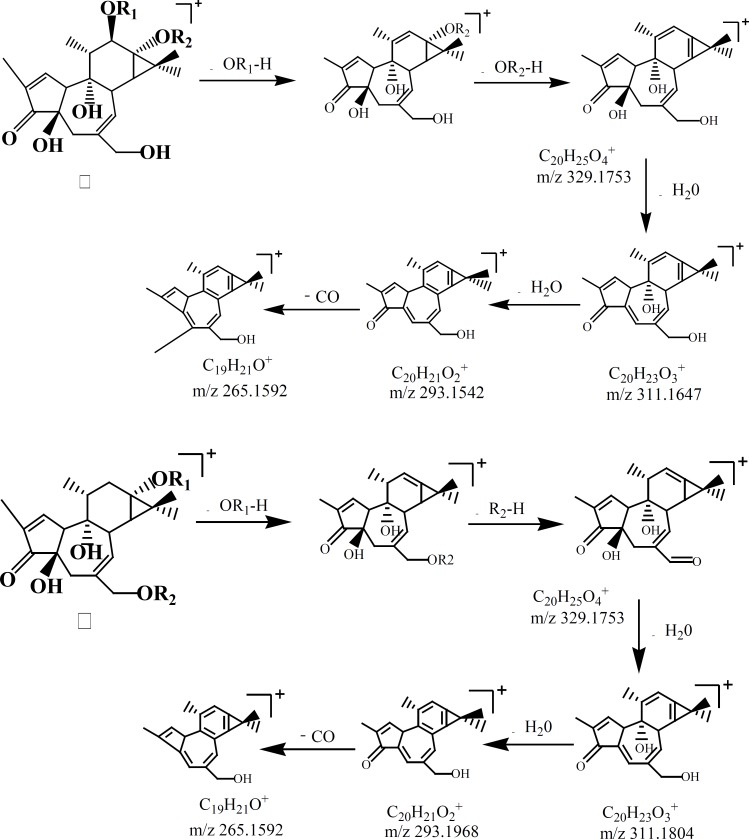
Proposed fragmentation pathways and characteristic ions of Phorbol esters: (׀) phorbol 12,13-diesters; (׀׀) Deoxy phorbol-13,20-diesters.

*Possible compounds and fragmentation pathways of phorbol esters of SCTp and SCTe*

**Figure 3:**
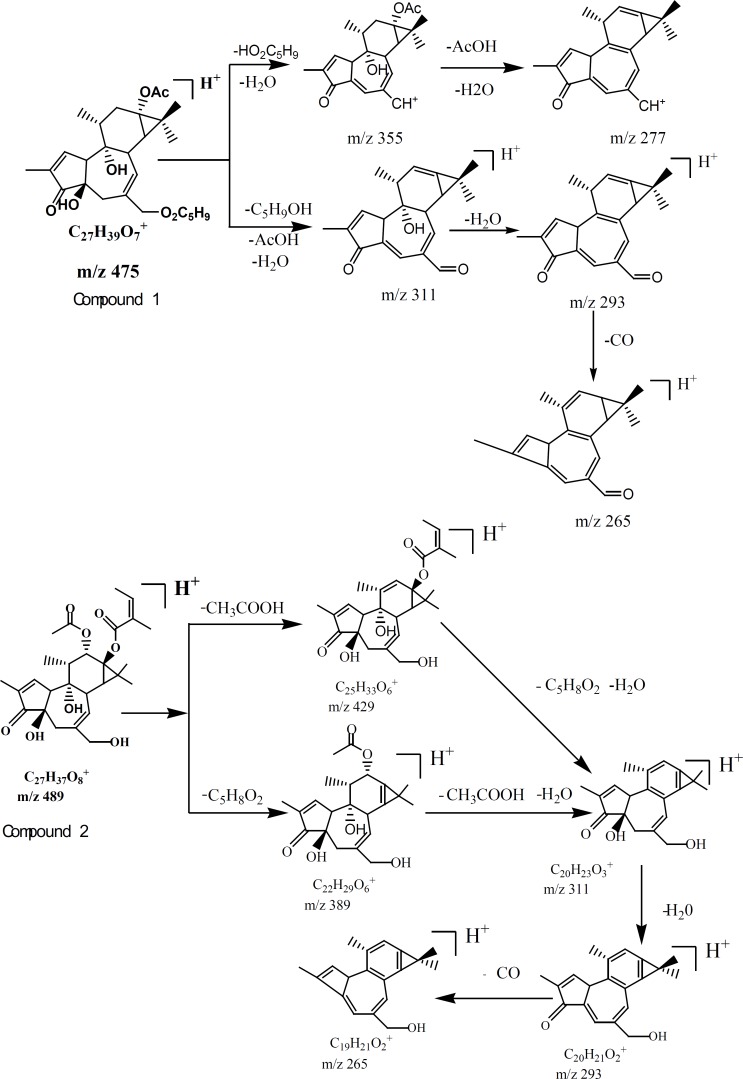
Proposed fragmentation pathways and characteristic ions of compound 1 and compound 2.

Six compounds speculated in SCTp and SCTe were summarized in Table 1 by HPLC-ESI(+)-MS. From the fragment ions of phorbol esters and the reported (28), as shown in Figure 2, two fragmentation pathways were proposed. The main fragments in the compounds were m/z 311(C_20_O_3_H_23_), 293 and 265, which was loss three molecules of water or organic acid (ROOH). In Figure 3, the fragmentation pathways of compound deoxyphorbol acetate methylbutanoate and phorbol acetate methylbutenoate were shown.

## Conclusions

In the present study, we evaluated the antinociceptive effect of SCT through the writhing test in mice, investigated the effects of it on spontaneous smooth muscle contractions of isolated rabbit jejunum, and examined the results *in-vitro* through the small intestine propulsion *in-vivo* and the possible compounds were detected in the active parts.

Acetic acid-induced writhing is a standard, simple, and sensitive test for measuring analgesia and has long been used as a screening tool for the assessment of analgesic or anti-inflammatory properties of plant extracts and natural products. It has been suggested that acetic acid acts by releasing endogenous mediators that stimulate the nociceptive neurons (29). In the present writhing test, there was a little reduction in writhing for the groups treated with SCTm, SCTp and SCTe; the inhibition were lower than 50% (Table 2). Although an analgesic pyrazine derivative isolated from the leaves of *C. tiglium* could remarkably inhibit the acetic acid-induced abdominal writhing in mice (7). From the results, SCT may decrease the abdominal pain in some traditional Chinese prescription, such as Wei-Chang-An-Wan (30). However, in this model, it is postulated that the abdominal constriction response is induced through local peritoneal receptor activation and involves prostanoids mediator (31). Additionally, an important disadvantage of this model is that the other classes of drugs can reveal the effect, such as adrenergic antagonists and muscle relaxants, favoring possible false positive results (21). Due to these reasons, the results from the writhing test should be for further investigation with other tests, such as hot plate test and formalin test.

In our previous work, it was demonstrated that Croton oil possessed spasmogenic and spasmolytic properties and the regulatory effects of Croton oil on gastrointestinal motility were mediated via the activation of M_3 _muscarinic receptor and Ca^2+^ influx through L-type Ca^2+^ channel (6). In this experiment, the effect of Croton oil and SCTm on smooth muscle contractions was compared. Through further investigated, the different parts of SCTm and both of SCTp and SCTe parts showed contract intestinal tissue, and conversely, *n*-BuOH parts and H_2_O fraction had the action of relaxing intestinal tissue. These findings suggested that *C. tiglium* itself possess a biphasic action. SCTp and SCTe had the same effect on gastrointestinal motility as Croton oil, which possessed spasmogenic properties at low concentration (0-0.06 or 0-0.04 mg/mL) and spasmolytic properties at high concentration (0.06-0.1 or 0.04-0.06 mg/mL). In the *in-vivo* study, SCTm at dose of 50, 100 and 200 mg/Kg increased the intestine propulsion in a dose-dependent manner, however, at dose of 200, 400 and 800 mg/Kg, the inhibitions of the intestinal propulsion were lower (data was not shown). SCTp and SCTe exhibited higher increasing in the intestinal propulsion (Table 3). Both of *in-vitro* and *in-vivo* results suggested that *C. tiglium* possessed spasmogenic and spasmolytic properties at different doses, and SCTp and SCTe were the active parts. So in the following work, we proposed the possible compounds in SCTp and SCTe by LC-MS.

It was reported that phorbol esters were the main active compounds in* C. tiglium*, and they were known as the activator of protein kinase C (PKC). PKC activation mediated various signaling pathways critical for the formation, regulation, and maintenance of the gastrointestinal tract (32). Phorbol esters could induce rapid, sustained contraction in smooth muscle cells isolated from guinea pig intestine and the contraction of phorbol was related to Ca^2+ ^(19, 20). The mechanism of Croton oil induced contraction was mediated via Ca^2^+influx through L-type Ca^2+^ channel (6). So, the active compounds for *C. tiglium *induced muscle contraction in isolated rabbit jejunum maybe phorbol esters.

By HPLC-ESI-MS/MS, six compounds (Table 1) were speculated from the SCTp and SCTe of *C. tiglium*. In Figure 2, we proposed the possible fragmentation pathways of phorbol esters. The characteristic fragment ions were m/z 311, 293 and 265. In LC-MS, side chains of phorbol esters such as fatty acid and H_2_O could be eliminated to give a stable fragment ion. To obtain a similarly stable fragment ion, only two esters/hydroxyl groups of deoxyphorbol esters were eliminated as free acid or water. The fragment ions of phorbol esters in the MS-MS mode were m/z 311→293, and a characteristic elimination of 28 u (-CO) from the fragment m/z 293 could be observed, thus, m/z 293 →265 could be detected (28).

In conclusion, the effects of muscle contraction and analgesic of SCT extracts both *in-vitro* and *in-vivo* were investigated and the compounds were analyzed in this experimental study. The results demonstrated that SCTp and SCTe could contract the smooth muscle and six phorbol esters were detected from them. However, the chemical compounds and the exact mechanisms of contraction must be further investigated.
